# The timeline of non-vocal and vocal communicative skills in infants with hearing loss

**DOI:** 10.3389/fped.2023.1209754

**Published:** 2024-01-12

**Authors:** Amanda Saksida, Roberta Rebesco, Arianna Colombani, Sara Pintonello, Eleonora Tonon, Andrea Martina Santoro, Eva Orzan

**Affiliations:** Institute for Maternal and Child Health-IRCCS “Burlo Garofolo”, Trieste, Italy

**Keywords:** communicative skills, language development, hearing loss, vocal skills, non-vocal skills, video analysis

## Abstract

**Objective:**

The study investigates what is the link between early verbal and non-vocal abilities, when does predominantly verbal communicative style occur after the intervention with cochlear implants (CI) or hearing aids (HA), and how predictive it is of later linguistic development in deaf and hard of hearing (DHH) infants and children.

**Methods:**

Cohort: children with moderate-to-profound hearing impairment (*N* = 49, 20 girls, mean age at HA or CI intervention = 15 months, range: 4–35 months).

**Measures:**

Receptive and productive vocabulary at 24 and 36 months and video analysis at 12 months post-intervention. Analysis: Predictive values of total and verbal responses to communicative turns for later vocabulary development were assessed, as well as the relative time course of the development of vocal/verbal communication in DHH children.

**Results:**

Vocabulary at 24 months is predicted by auditory responses at 12 months, as well as by overall responsiveness before intervention. Non-vocal responses decline and overall verbal responses increase significantly between 6 and 12 months after intervention. The trend is delayed in children with delayed (>12 months of age) treatment with CI or HA.

**Conclusions:**

Age of intervention affects the development of vocal/verbal communicative style. Language development, in particular, vocabulary growth, can be further stimulated by the enhancement of preverbal (both vocal and non-vocal) communicative skills.

## 1 Introduction

The development of early vocal and non-vocal communicative skills is strongly related to the later language outcomes and constitutes the base for later verbal communication. Deaf and hard of hearing (DHH) infants of hearing parents have an overall poorer experience of two-way interaction, compared to their hearing peers (1). Therefore, predominantly verbal communicative style—the ability to communicate in a self-preferred manner using predominantly verbal communication—may develop with delay in DHH children. The overall purpose of this study is to (1) investigate the link between early verbal and non-vocal abilities, (2) to understand when predominantly verbal communication style is achieved after intervention (INT) with cochlear implants (CI) or hearing aids (HA), (3) to extract the time course and potential delays in verbal communicative skills in DHH children, and (4) to assess how predictive it is of later language development.

Before the onset of speech production, infants communicate with adults through vocal acts [cries, vocalizations, and protophones (2, 3)], non-vocal acts (eye-contact, joint-attention episodes, motor responses, and gestures), and by combining the two modalities. Non-vocal acts constitute an important part of children's early communicative skills and have been proven to be, along with parents’ sensitivity and responsiveness to the child's requests, predictive of later language development (4–9). Soon after birth, infants are attentive to eye-gaze and interpret it as a communicative act; later on, joint attention abilities become predictive of language skills (10–12). In the second half of the year, they begin to use motor responses, such as reaching or handling, in communicative events (13). The first deictic gestures emerge around the same time and are mainly used by infants to guide the caregiver's attention or make requests. Around the emergence of the first word, infants start using representational and symbolic gestures, i.e., context-independent gestures used to represent objects, actions, or events, including conventional gestures such as shaking head to mean “no” or shrugging (9, 14, 15). From the onset of the first word, non-vocal communication automatically fades, and a great part of non-vocal acts become replaced by verbal acts (16). Non-vocal responses tend to disappear from children's communicative repertoires by the end of their second year, being substituted by predominantly verbal communication (17).

DHH children of hearing parents have an overall poorer experience of two-way interaction, compared to their hearing peers. They are usually more interrupted during their communicative attempts (18), more requested to produce words rather than being engaged in conversation, and experience fewer and shorter moments of joint attention (19) and vocal turn-taking (20, 21). This is because hearing parents of DHH children tend to be less responsive (4) and more directive in their interactions (22, 23), compromising children's ability to maintain attention (23, 24) and giving less feedback on their communicative attempts [see (1, 25)]. Due to such impoverished interactions, DHH children born in hearing families are at risk of receiving less communicative and linguistic stimulation compared to their hearing peers (5). To develop spoken language and communication abilities, proper communicative stimulation is crucial.

In clinical practice, one method to monitor and sustain the development of DHH children's communicative skills and promote a positive parent-child interaction is the use of video analysis. The Tait video analysis was created and validated as a part of the Nottingham Auditory Implant Programme and provides professionals with brief recordings of a dedicated play session between a child and a caregiver in a semi-ecological (laboratory) or structured home setting. From the recording, vocal communication skills (auditory autonomy, and the awareness of the sound of speech; see (26, 27) and non-vocal communication skills (appropriate eye contact, conversational-style turn-taking, joint-attention episodes, as well as motor and gestural responses the) can be analyzed. The method is a reliable tool in the assessment (28–30) and monitoring (31) of the pre-verbal language skills in children with hearing loss, correlating well with other auditory and structural speech and language tests (32). Data obtained through video analysis showed a significant correlation between preverbal communicative skills and later language development. Specifically, preverbal measure of “auditory autonomy” (whether through vocalization or gestures (33), has been shown to be highly predictive of later speech perception performance and language development (26, 34) and speech identification performance in DHH children 3 years after cochlear implantation (33). In addition, preverbal communicative skills (both vocal and non-vocal) obtained 12 months after implantation were shown to be predictive of later performance on speech perception tasks (26). Importantly, these data also indicated that children who received a cochlear implant between 1 and 2 years of age switched to a predominantly auditory/oral mode of communication by 12 months post-implantation (31).

In our Institute for Treatment and Research, the video analysis technique has been used in the last years as a clinical measure of communicative development in DHH children, auditory awareness in particular, and regular feedback was provided to caregivers of these children. However, no systematic overview of the time course of vocal and gestural developments has been available for Italian, nor has it been validated whether the overall communicative responsiveness and the development of vocal communicative style are indeed a good predictor of language development in our clinical population. Here we therefore investigate the comparability of the clinical data collected in our institute with the previously reported studies and the time windows in which the major transition from gestural to vocal communicative style occurs in young DHH children. Children with profound to moderate hearing loss (HI), who received either CI or HA, but were otherwise comparable in their verbal and non-verbal communication skills, were included in the study sample.

## 2 Methods

### 2.1 Participants

The study included a cohort of 49 children (20 girls, 29 boys, age at intervention: mean = 15.02 months, SD = 7.15, range: 4.0–35.27) with moderate to profound hearing loss (HI). 32 children received sequential bilateral CI, 14 children with moderate HI were HA users, and 3 children received combined intervention (CI + HA). 17 children received intervention between 15 and 35 months of age, while 32 children received it prior to 15 months of age. The anonymized raw dataset, including anamnestic data, is available in Supplementary Material S1 (Supplementary Table S1).

All children were longitudinally followed at the institute for at least 24 months after the intervention and had at least three video analyses recorded and analyzed. All children had normal cognitive abilities and motor skills [assessed as a part of clinical follow-up at 1 year after implantation with the Bayley Scales of Infant and Toddler Development, Third Edition (BSID-III)], and no uncorrected visual deficits. The moderate-to-profound non-syndromic sensorineural HI diagnosis had been made in the first months of life after a failed newborn hearing screening. The candidacy for CI surgery had been made after a period of traditional hearing aid amplification with limited or no benefit. All participants received the first CI or HA before 35 months of age; for CIs there was a variable interval between the first and second surgery. The average age of first HA fitting for children who later received CI was 8.2 months (SD = 6.8), and 12.3 months (SD = 6.8) for children with moderate HI. The average age for the first CI surgery was 16 months (SD = 7.2) (see Supplementary Table S1 for details). Both children with CI and with HA were regularly followed by the audiologist and speech therapists, and their mean-aided threshold was always fitted, following medical prescription, to ≤35 dB HL for the better ear, tested at the standard frequencies for the pediatric population (250/500–4,000 Hz). Their language comprehension scores (assessed as a part of clinical follow-up at 1 year after implantation using a combination of a picture-naming task and Child Development Inventory scales (35, 36) ranged from 5° to 90°. None of the children were bimodally bilingual (raised in oral and sign language simultaneously). Prior to data analysis we analyzed possible differences between the two subgroups regarding their auditory capacities, communicative abilities as assessed with the systematic video analysis, and language assessment outcomes. The final size of the group was limited by the number of children who met the above criteria, and that we had access to at the medical institute where the study was conducted. Participants came from various regions in Italy and had diverse socio-economic backgrounds.

Prior to the recordings, parents gave their written consent to participate in the recordings. They were also informed about the usage of clinical data for research purposes and gave their written consent to participate before the assessment (see Supplementary Material S3). The study was conducted in accordance with the 1964 WMA Helsinki declaration and its later amendments, under the framework of the research project RC 42/22 approved by the institutional ethical review board, nominated by the Italian Ministry of Health (Ufficio per la Ricerca Clinica IRCCS Burlo Garofolo).

### 2.2 Video analysis

If recording at home, parents were instructed to set up a static environment, behind a table or on the floor, and conduct a semi-structured interactive play with the child. The proposed activities were typically book reading, role play, or construction games. Following the procedure created and validated by Tait as a part of Nottingham Auditory Implant Programme, 20 communicative turns from a randomly selected central portion of the recording (the beginning and the end were never analysed) were analyzed from each recording (26, 27). Communicative turns were categorized as looking when the child could see the adult and could infer about the meaning of the turn by observing the scene, while turns in which the child had to rely on hearing were categorized as non-looking. Child's responses were categorized as vocal or non-vocal, or missing (null responses). A deictic or an iconic gesture, but also a head turn, an eye-contact, a playful movement of a toy, and reaching for/handing an object were categorised as non-vocal communicative turns whenever they represented a response to a conversational turn or its initiation. A non-verbal vocalization (a precanonical vowel-like sound, for example), if integrated in the relational context of dyadic conversation, was categorised as vocal turn. Laughs were not counted as vocalizations but as non-vocal turns in case they constituted a conversational turn. Turns that were both vocal and non-vocal were coded as vocal, because one of the main scopes of the Tait video analysis technique is to observe the vocal development in deaf populations. The final scores for the following variables were expressed as percentages of a total 20 turns: vocal looking, vocal non-looking, non-vocal, and no response. Communicative turns can furthermore be categorized as autonomous if the communicative initiative is taken by the child. The latter variable was, however, not consistently coded in the clinical practice for our dataset.

### 2.3 Language assessment (PVB, Ping, TFL)

Three assessment tools were used to evaluate receptive and productive vocabulary in the study sample. For the assessments from 8 to 36 months of age, the Italian version of the MacArthur–Bates Communicative Development Inventories (MB-CDIs) (35) called PVB_Primo Vocabolario del Bambino test (36), was used. The questionnaire is used for the assessment of linguistic development, from non-verbal components to grammatical development, accurate and valid in children with typical development as well as in atypical populations, including deaf children with CIs. Additionally, a direct language observation was conducted using the standardized test PinG Parole in Gioco (18–36 m.o.). The test was used to evaluate the comprehension and lexical production of nouns along with the understanding and production of predicates and consists of a series of color images with names or predicates that must be comprehended by pointing and naming. The Test Fono Lessicale (TFL) (37) (>30 m.o. for receptive and >36 m.o. for productive vocabulary) was administered to further assess the receptive and productive vocabulary. In all three assessment tools, the results were compared to the standardized scores, and percentile ranks were measured for each test administration.

### 2.4 Procedure and statistical analysis

Data for all children were collected and analyzed retroactively from the existing medical reports. For children that were regularly followed at the institute in the last few years, we collected the basic demographic data and the following clinical variables: age at intervention (INT) with hearing aids or cochlear implants, mean-aided threshold at 0 and at 12 months post INT, receptive and productive vocabulary scores at 0, 6, 12, 24, and 36 months post INT, the results of the video analysis at 0, 6, 12, and 24 (if existing) months post INT. From the video analysis, the following measures were considered: non-vocal and null responses, looking vocal turns, and non-looking vocal turns (auditory awareness). Total vocal responses were computed by summing all vocal turns. For each, the proportion of 20 communicative turns were computed.

We have first computed regression models to assess whether the results of video analysis at 0 and at 12 months post INT were predictive of vocabulary development at 24- or 36-months post INT. The point in time when vocal responses prevail, and non-vocal responses decrease in frequency was assessed in each of the two subgroups by using the two linear regression models and calculating the intersection point between the two regression lines. To estimate the average point of intersection, the two models and their intersection were repetitively (*N* = 500) calculated for randomly selected subsets of the main data set. The average intersection points for the two groups were compared using the nonparametric Wilcoxon-test. The anonymized raw dataset is available in Supplementary Material S1 (Supplementary Table S1).

## 3 Results

Initially we analysed the differences between the two subgroups regarding their auditory threshold, communicative abilities as assessed with the systematic video analysis, and language assessment outcomes, using a series of Wilcoxon rank sum tests given the non-normal distribution of the variables. At intervention, infants with CI had higher auditory threshold, as measured with pure tone audiometry (PTA) (W = 336.5, *p* = 5.54×10^−05^). Later on, at 6 and 12 months post INT, there were no significant differences between the two subgroups in their PTA thresholds. At 12 months post INT, children with HA exhibited somewhat lower percentage of vocal (ME = 0.34; W = 226.5, *p* = 0.043) and higher percentage of non-vocal responses (ME = 0.4; W = 87.5, *p* = 0.029) compared to children with CI (ME = 0.54 and 0.22, respectively). Again, this difference vanishes at 24 months post INT. Similarly, there were no significant differences in their Receptive and productive vocabulary at 24 or 36 months post INT. We therefore collapsed both subgroups for the subsequent analyses.

**Figure 1:**
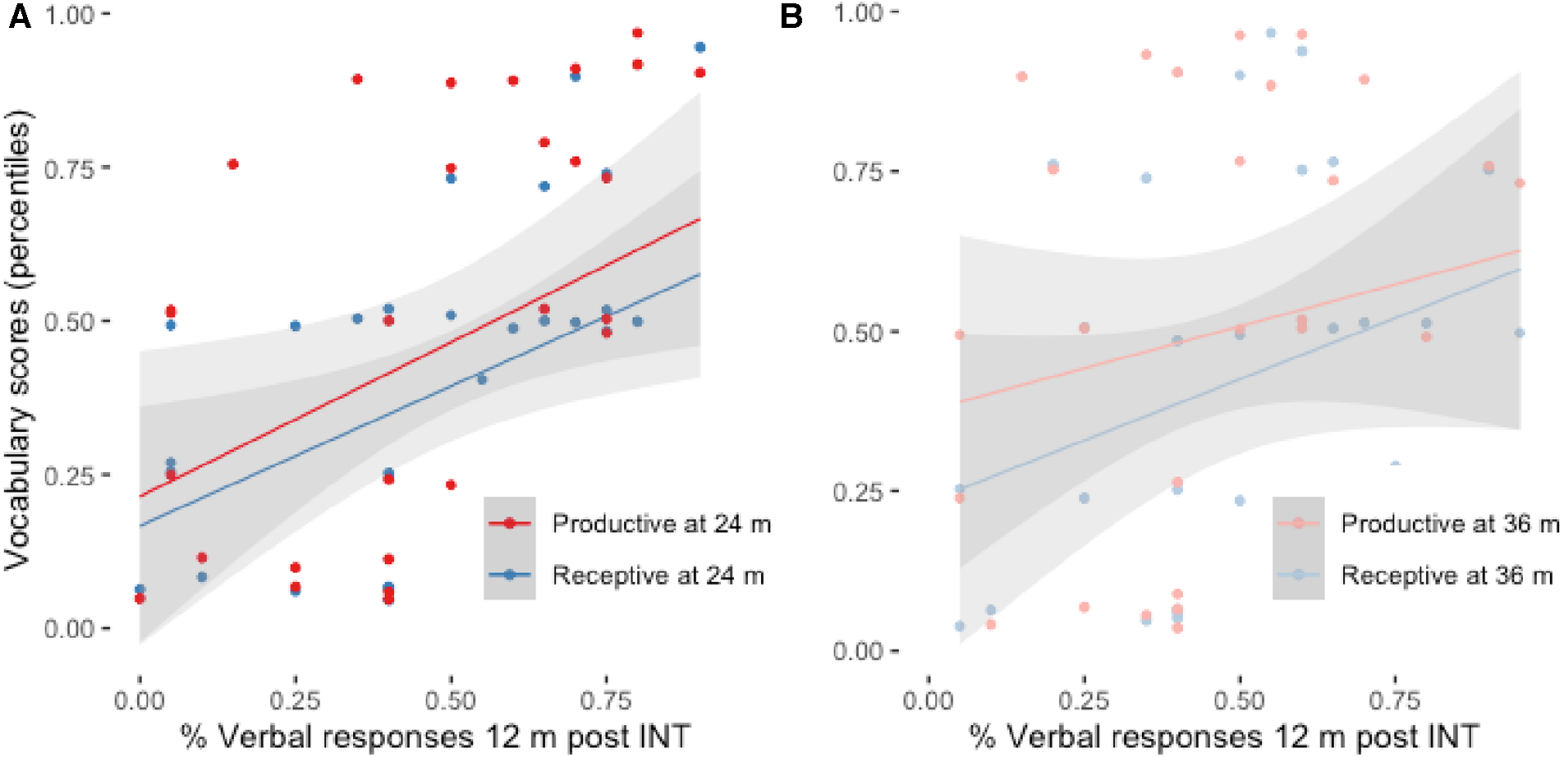
(**A**) Receptive and productive vocabulary at 24 months post INT as a function of verbal responses at 12 months post INT. (**B**) Receptive and productive vocabulary at 36 months post INT as a function of verbal responses at 12 months post INT. The grey areas represent std. error of the estimate for each linear regression line.

To assess whether predominantly vocal and auditory-based communicative behavior is predictive of speech perception skills later in development also in our sample of children [cf. (26, 32)], we created simple linear regression models with Receptive and Productive vocabulary scores at 24 and 36 months as outcome variables, and Vocal responses to communicative turns at 12 months after the intervention with CIs or HA (INT). The analysis of variance of the models showed a significant effect of Vocal responses at 12 months post INT for Receptive [M1: adj *R*^2^ = 0.1744, F(1, 28) = 7.127, *p* = 0.013] and Productive vocabulary scores at 24 months post INT [M2: adj *R*^2^ = 0.1293, F(1, 31) = 5.752, *p* = 0.023]. The scatterplots and the fitted regression lines are presented in Figure 1A. Although the trend is similar for vocabulary scores at 36 months, results of the models were not significant due to larger variability in the sample (Figure 1B).

**Figure 2:**
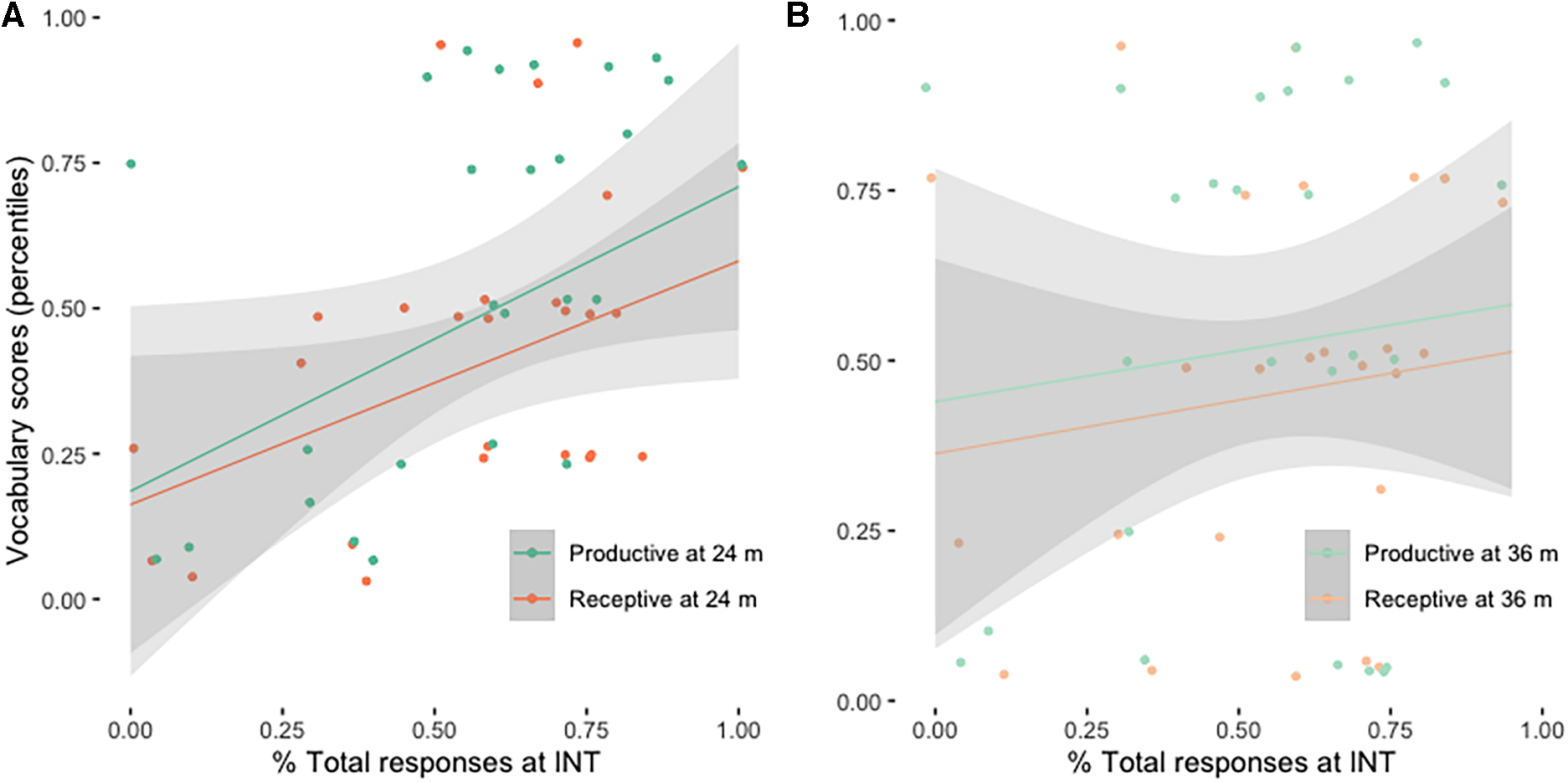
(**A**) Receptive and productive vocabulary at 24 months post INT as a function of total responses at the time of INT. (**B**) Receptive and productive vocabulary at 36 months post INT as a function of total responses at the time of INT. The grey areas represent std. error of the estimate for each linear regression line.

Communicative skills develop to some extent irrespective of hearing status, and it has been shown that overall communicative skills, both vocal and non-vocal, especially communicative autonomy, can have an impact on later language development [cf. (263)]. Given that our clinical data lacked systematic analysis of autonomy, we examined whether Total responses, both vocal and non-vocal, also predict later language development. Similar to the previous models, responses to communicative turns immediately before the INT were predictive of Productive [M3: adj *R*^2^ = 0.107, F(1, 28) = 4.457, *p* = 0.044] and marginally significant of Receptive vocabulary development at 24 months post INT [M4: adj *R*^2^ = 0.092, F(1, 25) = 3.627, *p* = 0.0683], but not at 36 months post INT (Figures 2A,B).

**Figure 3:**
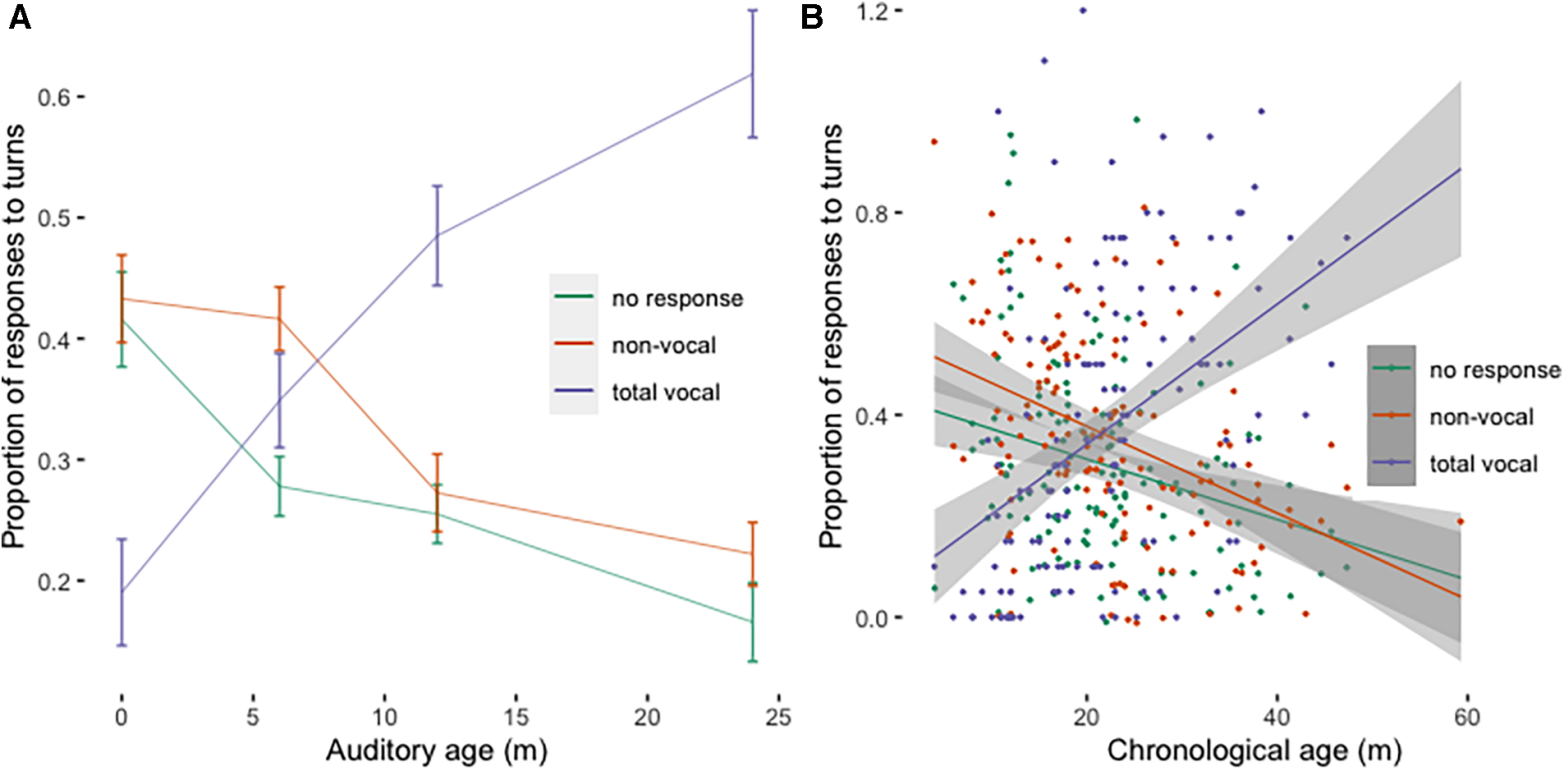
(**A**) Proportion of vocal/verbal, non-vocal, and no responses across time post INT. Error bars represent standard errors. (**B**) Proportion of vocal/verbal, non-vocal, and no responses across chronological age. The grey areas represent std. error of the estimate for each linear regression line.

For most children in our sample, vocal communication style prevailed over time. Conversely, the proportions of non-vocal and absent/no responses to communicative turns decreased in time. At 12 months post INT, children in our sample exhibited a relatively low proportion of auditory awareness (ME = 0.33, SD = 0.233) and the proportion of total vocal/verbal responses occurred in roughly half of communicative turns (ME = 0.485, SD = 0.26). Vocal/verbal responses become prevalent (i.e., more than 50%) in the whole group only 24 months post INT (ME = 0.619, SD = 0.21). In Figure 3, proportions of vocal, gestural and No responses as a function of time post INT, and as a function of chronological age, are presented. The linear regression models with Vocal or Non-vocal responses as outcome variables, Time post INT as the dependent variable, and subject variability as the random intercept, showed a significant increase in time post INT for Vocal (M5: *β* = 0.205, SE = 0.002, t = 8.224, *p* < 0.001), and a significant decrease in time post INT for Gestural responses (M6: *β* = −0.011, SE = 0.002, t = −5.316, *p* < 0.001). In both models, the variance among participants did not exceed the magnitude of the fixed effects β values. Visual inspection of residual plots for both models (Supplementary Figure S2) showed the predicted heteroscedasticity and low autocorrelation, indicating that the model is valid in predicting the effects. The summaries of the models are available in the Supplementary Material S2.

The point in time when vocal responses prevail, and non-vocal responses decrease in frequency was assessed in each of the two subgroups by using the two linear regression models and calculating the intersection point between the two regression lines. To estimate the average point of intersection, the two models and their intersection were repetitively (*N* = 500) calculated for randomly selected subsets of the main data set. According to this simulation, and as visible also from Figure 3, the average point at which we see the decrease of non-vocal responses and an increase of vocal/verbal responses is 21 months (SD = 3.601, range: 6–36 months) in chronological age, or 7.5 months post INT (SD = 2.654, range: 0–24 months).

### 3.1 Explorative analysis of the time-course of non-vocal and vocal responses

**Figure 4:**
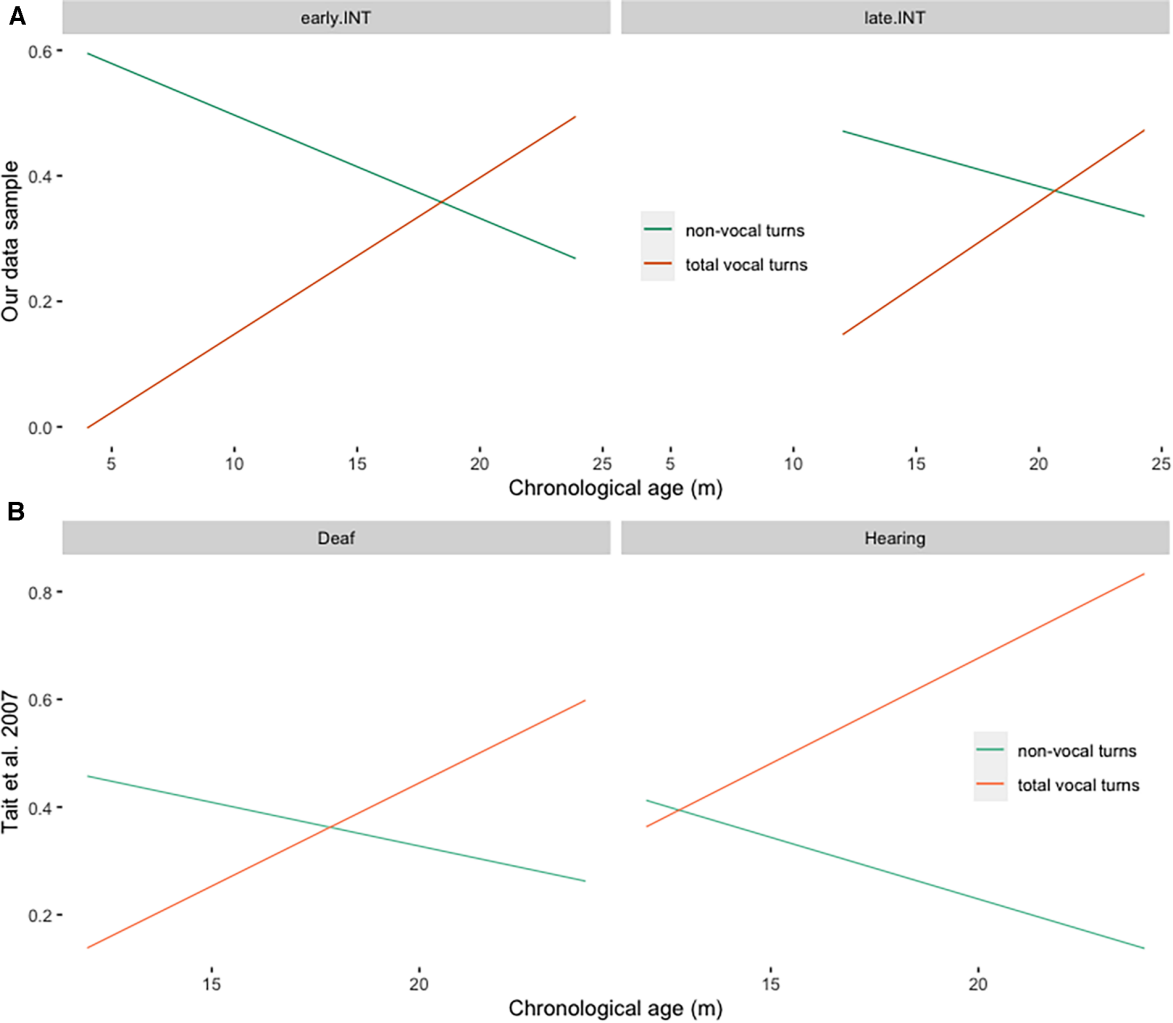
(**A**) Linear regression lines of vocal and non-vocal responses to communicative turns across time in our data sample, divided by the age of INT (early.INT = INT within 12 months, late.INT = INT after 12 months). (**B**) Linear regression lines of vocal and non-vocal responses to communicative turns across time in the sample of children in Tait et al. (39), divided by Group (Deaf = deaf children implanted before 12 months, Hearing = chronological age matched normally hearing children).

A question that arises upon the above analysis of the exchange between non-vocal and vocal responses to communicative turns during verbal development was whether it is comparable to other published data with DHH children, and how does this transition compare to normally developing children. To our knowledge, the only study that longitudinally observed the development of these communicative features in both DHH and hearing children using the same methodology is the study by Tait, De Raeve, & Nikolopoulos (38). They report longitudinal data from 10 deaf children implanted before 12 months of age, and 10 chronological-age matched children with normal hearing. We therefore compared their data on vocal and non-vocal responses over time with our data sample. To be able to directly compare the two studies, we subgrouped out data into children with CI or HA intervention before 12 months (*N* = 26), and those after 12 months (*N* = 23). The results, originally presented in data tables, are visualized as regression lines in Figure 4. In both studies, the transition between non-vocal and vocal responses occurs sometimes between 15 and 20 months for early rehabilitated DHH children (our sample, using the same procedure as above to compute the average intersection point: ME = 19 months, SD = 3.33), and is delayed in our sample of DHH children with late INT (ME = 22 months, SD = 5.52). The Wilcoxon signed-rank test of the difference between the intersection points calculated during the random subsampling for the two groups indicates that this delay is significant (W = 158200, *p*-value < 2.2×10^−16^). In the group of normally hearing children in the study by Tait et al. (38), this transition occurred somewhat earlier, between 12 and 15 months.

## 4 Discussion

Children in the present cohort were longitudinally followed at our Institute for Treatment and Research for at least 24 months after the INT and had at least three video analyses recorded and analyzed. Children's overall communicative abilities at INT and their ability to communicate vocally/verbally at 12 months post INT are predictive of later vocabulary development, which is in line with previous findings (26, 39). Furthermore, in this sample, we observe a trade-off between non-vocal and vocal/verbal communicative styles somewhere before the second birthday, leading to the predominantly vocal/verbal communication later in development. Again, such development conforms with previously reported results on the difference between hearing children, DHH children with early INT, and DHH children with late INT (38). Indeed, the onset of predominantly vocal/verbal communication occurs early in the second year of life for hearing children, somewhere between 15 and 20 months in DHH children with early INT, and somewhere between 20 and 24 months in DHH children with late INT. Our results once more confirm that early auditory input is crucial for the development of predominantly vocal/verbal communicative style, and timely interventions are crucial for the development of communication styles similar to hearing children.

Moreover, the present results also show that overall communication skills early on, before INT, play an important role in later language development. Early non-vocal responses, i.e., eye-contact, joint-attention episodes, motor responses, and gestures, constitute a large part of early communication, importantly contributing to the development of efficient communication. Our results are thus in line with previous literature showing correlations between early non-vocal communication and later language development, confirming an important role of non-vocal behaviors for later communication development also in the context of hearing loss. Taking these premises into account we hypothesize that clinicians could promptly intervene in the first phases of CI or HA fitting by taking advantage of DHH intact communicative modality (visual-non-vocal) to scaffold auditory-verbal language development, thus supporting overall language development at the very earliest stages.

There are, however, several points that remain open with the present study. In the studies that applied the Tait video analysis method that has been developed explicitly for the evaluation of overall communication skills in DHH infants and children, including ours, the proportions of non-vocal communicative acts are considerably higher than those reported elsewhere (40). This difference comes from the fundamental difference in coding: in our study, we counted all non-vocal communicative acts, not just iconic or deictic gestures. In fact, most of the non-vocal acts in our datasets consists of motor responses such as handling, reaching, head-turning, and nodding (59%), or eye-contact (22%), while only 19% of acts were deictic or symbolic gestures. The results of the present study indicate that all non-vocal communicative acts, not only gestures, are a prerequisite for successful development of verbal communication later in development.

Throughout the work we have considered exclusively communicative vocalizations and gestures while omitting all other non-communicative acts, either vocal or non-vocal. We have therefore no insight into the rate with which children with hearing impairment may produce either non-communicative gestures or vocalizations in the form of protophones. Children in our sample also have overall lower scores in auditory awareness and total vocal responses compared to the results reported by previous studies (26, 32). It is possible that the trade-off between non-vocal and vocal/verbal communication is to some extent language specific, and that it is therefore difficult to compare data from different languages (41). Another possibility is that children in other studies were exposed to different rehabilitation methods that enhanced responsiveness to a greater extent compared to our sample.

Furthermore, we lack systematic collection of data on verbal autonomy (i.e., child's initiative in communication) and data on joint attention during communication. These aspects of preverbal communication have been shown to significantly predict later language development (31).

Lastly, the analysis of the present dataset showed overall congruence with the previous findings. Nonetheless, an important proportion of children in our sample are reported to have good vocal/verbal communication but relatively low vocabulary scores (Figure 1, points below grey areas). On the one hand, our outcome variables only consist of vocabulary outcomes, while language and communication engage several domains that do not only refer exclusively to vocabulary. On the other hand, the existing analysis of the recorded videos, which considers the proportion of looking and non-looking vocal/verbal responses to communicative turns but does not take a closer look into the quality of the responses (how meaningful and appropriate an answer is in a given communicative situation), might miss some aspects of communicative and verbal development, especially in DHH children. A similar consideration is possible for the quality of conversational turns observed during the video analysis: a child may have, in a given context, repeated the same vocal or non-vocal response many times in the randomly selected portion of the conversation. Regardless of its quality, it is nonetheless a communicative act and should be treated as such. If anything, if such non-variable interaction is persistent, it tells more about the communicative skills of the adult in the diad than about communicative abilities of the child. While adult communicative style has not been assessed in the present study, it represents a valuable piece of information in clinical evaluation. It is therefore possible that a more detailed video analysis, one that takes all aspects of communicative turns into account, might give a better insight into children’s early communicative abilities, parental communicative style, and better predict later language development.

## Data Availability

The original dataset presented in the study is included in the Supplementary Material S1; further inquiries can be directed to the corresponding author.
