# Supplementary material for: The timeline of non-vocal and vocal communicative skills in infants with hearing loss
Source: Front Pediatr. 2024 Jan 12;11:1209754. doi: 10.3389/fped.2023.1209754 (PMC10811201; doi:10.3389/fped.2023.1209754)
Supplement: Supplementary file 2 [file Table1.docx]

Supplementary Material S1

The timeline of gestural and vocal communicative skills
in infants with hearing loss

Roberta Rebesco^1**^, Amanda Saksida^1**^, Arianna Colombani^1*^, Sara Pintonello^1^, Eleonora Tonon^1^, Andrea Martina Santoro^1^, Eva Orzan^1^

^1^﻿Institute for Maternal and Child Health-IRCCS “Burlo Garofolo” – Trieste, 34137 Italy

** first authorship
***** Correspondence: Arianna Colombani
colombaniarianna@gmail.com

# Consent form
