# Supplementary material for: The timeline of non-vocal and vocal communicative skills in infants with hearing loss
Source: Front Pediatr. 2024 Jan 12;11:1209754. doi: 10.3389/fped.2023.1209754 (PMC10811201; doi:10.3389/fped.2023.1209754)
Supplement: Supplementary file 3 [file Table2.docx]

**Supplementary material S2**

M1 <- lm(Receptive.24 ~ VANtotalverbal.12, data=outcomes)

M2 <- lm(Productive.24 ~ VANtotalverbal.12, data=outcomes)

M3 <- lm(Receptive.24 ~ VANtotal.00, data=outcomes)

M4 <- lm(Productive.24 ~ VANtotal.00, data=outcomes)

M5 & M6

data_early=outcomes.melt[outcomes.melt$age.range=="early.RE",]

data_late=outcomes.melt[outcomes.melt$age.range=="late.RE",]

n=nrow(data_early/data_late)

nt=0.2*n; neval=n-nt; rep=50; set.seed(123456789)

interceptx_early/interceptx_late=dim(rep)

subsett.a=NULL

for (k in 1:rep) {

tryCatch({

subset=sample(1:n,nt)

subsett.a <- rbind(subsett.a,subset)

M5 <- lm(VAN.total.verbal ~ age, data=data_early/data_late[subset,])

M6 <- lm(VAN.gestural ~ age, data=data_early/data_late[subset,])

interceptx_early/interceptx_late[k] =

abs((coef(rep.v)[1] - coef(rep.g)[1])/(coef(rep.v)[2]-coef(rep.g)[2]))

}, error=function(e){cat("ERROR :",conditionMessage(e), "\n")})

}

interceptx_early

interceptx_late


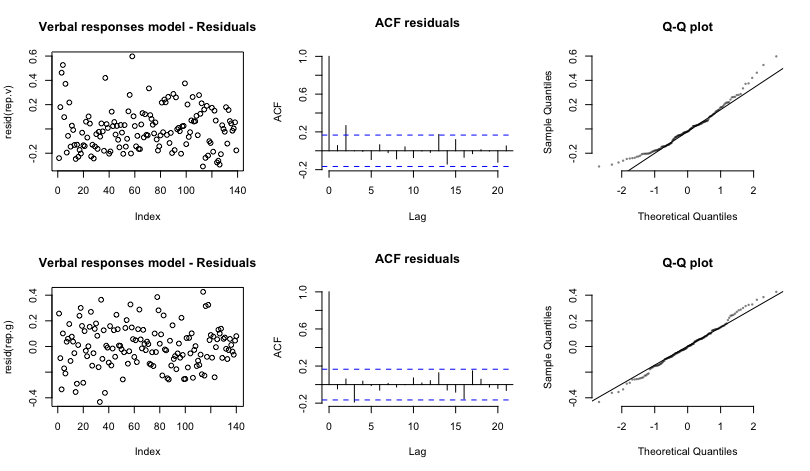


Figure S2. A Correlation plot of the residuals of the models M5 and M6. B Autocorrelation plot of the residuals of m1. C Q-Q plot of the residuals of the initial model.
